# New Oncologic Drugs from 2008 to 2023—Differences in Approval and Access between the United States, Europe and Brazil

**DOI:** 10.3390/curroncol31080332

**Published:** 2024-08-02

**Authors:** Rafael Balsini Barreto, Andressa Moretti Izidoro, Mario Henrique Furlanetto Miranda

**Affiliations:** Centro de Pesquisa Oncológicas—CEPON, Rodovia Admar Gonzada, 655, Florianópolis 88034-000, SC, Brazil

**Keywords:** Agência Nacional de Vigilância Sanitária (ANVISA), Food and Drug Administration (FDA), European Medicines Agency (EMA), regulatory review times, cancer drugs, drug approval

## Abstract

Introduction: Advancements in oncology have revolutionized cancer treatment, with new drugs being approved at different rates worldwide. Our objective was to evaluate the approval of new oncological drugs for solid tumors by the Food and Drug Administration (FDA), the European Medicines Agency (EMA), and the Brazilian Health Regulatory Agency (ANVISA) since 2008. Methods: Data were collected from public and online databases by searching for the date of submission, the date of the procedure, the date of approval, clinical indication, and drug characteristics. The distribution was tested using the Shapiro–Wilk, test and comparisons were made using the Mann–Whitney U test; the data are reported using median days and interquartile range (IQR1–IQR3). Results: In total, 104 new oncologic drugs for the treatment of solid tumors were approved by the three agencies: 98 by the FDA, 90 by the EMA, and 68 by ANVISA. The cancer types with the highest number of first indications were lung cancer (n = 24), breast cancer (n = 15), and melanoma (n = 15). Most approvals were for oral medications (n = 63) and tyrosine–kinase inhibitors or other small-molecule inhibitors (n = 54). Time to approval after submission was as follows: the FDA—224 days (167–285); the EMA—364 days (330–418); and ANVISA—403 days (276–636) (*p* < 0.00001 for the FDA to the EMA and the FDA to ANVISA). The difference between submission dates among the agencies was as follows: EMA–FDA: 24 days (0–85); ANVISA–FDA: 255 (114–632); and ANVISA–EMA: 260 (109–645). The difference in approval dates between the agencies was as follows: EMA–FDA: 185 days (59–319); ANVISA–FDA: 558 (278–957); and ANVISA–EMA: 435 days (158–918). Conclusions: New oncologic drugs are submitted to the FDA and EMA for approval on similar dates; however, the longer appraisal period by the EMA pushes the approval date for Europe to approximately 6 months later. The same steps at ANVISA delay the approval by 1.5 years. Such procedures cause a significant difference in available medications between these regions.

## 1. Introduction

The approval of numerous drugs has provided significant advancements in oncological treatment in the last 15 years, thus revolutionizing the management landscape for various types of cancer and providing hope for patients. The discovery of new targets and strategies to circumvent resistance mechanisms, coupled with innovation in drug development technology, is the key factor for such progress [1,2].

From 2019 to 2023, the United States Food and Drug Administration (FDA) approved 235 new drugs, excluding vaccines, diagnostic agents, and biosimilars, of which 59 (25.10%) are utilized in the treatment of solid and hematologic malignancies, both in adults and children. Similarly, in the same timeframe, the European Medicines Agency (EMA) approved a comparable number of new drugs: 181 new drugs with 53 aimed at utilization in the treatment of cancer (29.28%). Within this 5-year interval, 70% of the drugs approved in the United States received first approval in this region [3,4].

Accessing historical data on the Brazilian Health Regulatory Agency’s (ANVISA, abbreviated from the Portuguese “Agência Nacional de Vigilancia Sanitária”) approvals is challenging due to the scarcity of a publicly and easily accessible database; however, over the past two years (March 2022 to March 2024), Brazil has seen the emergence of 37 new drugs, 15 of which are anticancer agents, representing 40.54% of the total number of approvals [5].

The drug approval process is similar among the three aforementioned agencies: the process involves the submission of the dossier by the applicant, a period of analysis by experts, receipt of the report by other committee members, a new round of questions for the applicant if necessary, and, lastly, the final recommendation with the final label. All of these steps are taken to ensure safety when translating the information from clinical trials to the general population.

To hasten the review and approval processes, and consequently bring drugs to market as swiftly as possible, health agencies offer expedited pathways, such as fast track, breakthrough therapy designation, priority review, and accelerated approval for the FDA, in addition to accelerated assessments, conditional marketing authorization, approval under exceptional circumstances, and priority medicines for the EMA [6].

ANVISA is pursuing a similar initiative, albeit with fewer expedited programs. Since late 2017, the agency has introduced a special registration pathway for rare diseases and orphan drugs aimed at shortening official review times, based on Law #13411 from December 2016 [7], and started participating in Project Orbis, led by the Oncology Center of Excellence at the FDA [8].

Recognizing that delays of weeks in the initiation of oncological treatment, both in the adjuvant and metastatic settings, can impact overall survival [9], it is reasonable to conclude that delays in the approval and availability of new drugs depending on where a person lives can impact the lives of thousands of patients [10]. With this in mind, we opted to conduct original research aimed at identifying differences in approvals over the last 16 years within the United States, Europe, and Brazil.

## 2. Methods

### 2.1. Search and Selection Criteria

We systematically identified all newly approved oncological drugs and biological agents by the FDA and EMA from 1 January 2008, to 31 December 2023, spanning a period of 16 years.

To achieve our research aims, our methodology entailed the utilization of online databases accessible to the public provided by the respective regulatory agencies detailing drug approvals. With regard to the FDA, we relied on the annual reports issued by the Center for Drug Evaluation and Research concerning new molecular entities and original biological approvals. Similarly, for the EMA, we consulted the annual reports published by the Committee for Medicinal Products for Human Use, focusing on the identification of novel active substances [11,12]. As this search process provided us with information on all new drugs approved for any disease with their every indication, we excluded from our analysis all drugs lacking anticancer properties, generic and biosimilar drugs, and those initially indicated solely for hematological malignancies such as lymphomas, leukemias, multiple myeloma, and myelodysplastic syndromes.

ANVISA provides readily available information of this nature solely for the preceding 24 months; thus, we were unable to perform the same search within ANVISA’s database. Consequently, our approach involved the compilation of every approved drug by the FDA and/or EMA by cross-referencing their approval status with ANVISA.

Approval statuses of all expedited program applications were encompassed within our study.

We opted for a 16-year timeframe as this timeframe aligns with the accessibility of public data from health regulatory agencies.

### 2.2. Data Collection

Utilizing the FDA’s first letter of approval available via the DRUGS@FDA website, the EMA’s European Public Assessment Report for the initial marketing authorization documents, and ANVISA’s procedural documentation for new product registration [13,14,15], we extracted the following key information:

- Date of submission: the date at which the committees responsible for drug evaluation and approval received the documentation from the pharmaceutical industry;
- Date of procedure start: the date the committees started the evaluation;
- Date of approval: the date on which the committees issued the approval recommendation;
- Clinical indication: the first indication considering cancer primary site, associated biomarkers, and line of treatment;
- Drug characteristics: the drug’s class or mechanism of action and target, if applicable.

All drugs included and data collected underwent a double-checking process, and any discrepancies were solved through consensus.

The dates of submission, procedure start, and approval were collected from all agencies only if the drug received its first approval with an identical clinical indication. We employed such criterion understanding that different therapeutic indications are the product of different lines of research, so may involve submission to regulatory agencies in different periods with different materials, and therefore cannot be directly compared.

### 2.3. Data and Statistical Analysis

When conducting comparisons between the time to approval and the date of approval, only drugs approved for the first time with identical indications were included. We decided to use the FDA database as the baseline comparison as it is the agency that has provided the most approvals of new medicines in the last few years.

Descriptive statistics are utilized to report the dates, clinical indications, and drug characteristics. To assess the distribution normality of the time between submission and approval among the agencies, the Shapiro–Wilk test was utilized. Subsequently, comparisons were conducted using the Mann–Whitney U test in STATA 14.2 (StataCorp LLC, College Station, TX, USA). The results are presented in terms of median days and interquartile range (IQR1–IQR3).

## 3. Results

### 3.1. Approvals

From 2008 to 2023, 104 new oncological drugs for the treatment of solid tumors were approved by either the FDA, the EMA, or ANVISA. Specifically, the FDA approved 98 drugs, the EMA approved 90 drugs, and ANVISA approved 68 drugs. The complete list of drugs and their respective approvals is provided in Table 1.

The medications approved by the FDA but not the EMA for the same first indication include erdafitinib, tazemetostat, lurbinectedin, margetuximab, infigratinib, bezultifan, mobocertinib, tisotumab vedotin, mirvetuximab soravtansine, retifanlimab, toripalimab, fruquitinib, repotrectinib, and capivasertib. Among these drugs, erdafitinib and mobocertinib were approved by ANVISA.

In completing our search, we found a few drugs approved by the EMA but not approved by the FDA for the same indication as their first approval, and they include mifarmutide, catumaxomab, vinflunine, tegafur/gimeracil/oteracil, nintedanib, and padeliporfin. Of these drugs, mifarmutide, vinflunine, and nintedanib were approved by ANVISA.

Notably, we were unable to identify any drug approved by ANVISA that was not approved by either the FDA or the EMA.

The neoplasm with the highest number of first indications for these drugs was lung cancer, accounting for 24 out of 105 (23%), followed by breast cancer (n = 15–14%), melanoma (n = 12–11%), prostate cancer (n = 9–8%), and kidney cancer (n = 7–6%). All other primary sites had fewer than five new drugs approved for their treatment (see Table 2).

Most of the approved drugs are oral medications (n = 63–61%) as opposed to injectables (n = 41–39%). Regarding their class, the target drugs constituted the most common class, including tyrosine–kinase inhibitors (n = 35–34%), other small-molecule inhibitors (n = 19–18%), monoclonal antibodies, and antibody–drug conjugates (n = 27–26%). Regarding more traditional classes, there were eight new cytotoxic agents and seven new drugs targeting hormonal modulation (see Table 3).

We identified 89 drugs with specific targets, with the most common being anti-PD1, PD-L1, and CTLA-4, referred to as checkpoint inhibitors, comprising 13 new drugs. Other common targets include EGFR (n = 6–7%), HER-2 (n = 6–7%), BRAF or MEK (n = 6–7%), ALK (n = 5–6%), and VEGF/R, PARP, FGFR, and anti-androgens (n = 4–5% each). Additionally, there are eight drugs classified as tyrosine–kinase inhibitors with at least two targets (9%).

### 3.2. Submission Dates

Considering drugs that were submitted to at least two regulatory agencies, 19 of them were first submitted to the EMA, whereas 2 were simultaneously submitted to both the FDA and the EMA. Only one was first submitted to ANVISA (lutetium (177Lu) oxodotreotide)). The remaining drugs were first submitted to the FDA.

The median difference in submission dates was 24 days (0–85) when comparing data for the FDA to the EMA, 255 days (114–632) when comparing data for the FDA to ANVISA, and 260 days (109–645) when comparing data for the EMA to ANVISA.

**Figure 1:**
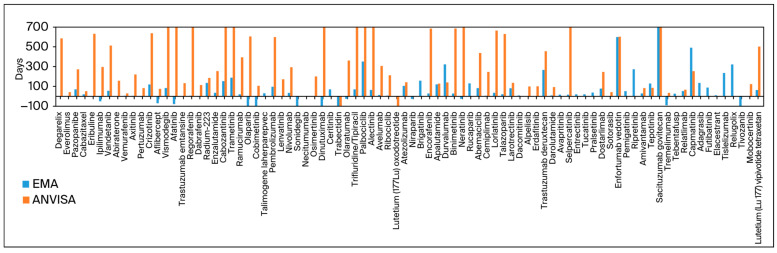
Difference in submission date in days compared to that of FDA submissions as the baseline.

Figure 1 exhibits all drugs approved by the FDA and at least one other agency, with the columns representing the difference in days between submission dates. The FDA was selected as the baseline because it approved the most drugs and was the primary agency for initial submissions in most cases.

### 3.3. Analysis Time

In order to calculate the analysis time, we used the difference between approval and submission dates. The FDA took a median of 224 days (167–285) to approve a new drug after submission, whereas the EMA took 364 days (330–418) and ANVISA took 403 days (276–636). Using the Mann–Whitney U test, the *p*-value was <0.0001 for the comparison between the FDA and the EMA and the FDA and ANVISA. For the comparison between the EMA and ANVISA, the *p*-value stood at 0.3304.

Both the EMA and ANVISA provide the date on which the procedure starts in their approval reports, enabling us to calculate the time from industry submission to the commencement of agency analysis. The EMA took a median of 22 days (21–27) to start the analysis procedure, whereas ANVISA took 73 days (46–164). Using the Mann–Whitney U test, the *p*-value was noted as <0.0001 for this comparison.

**Figure 2:**
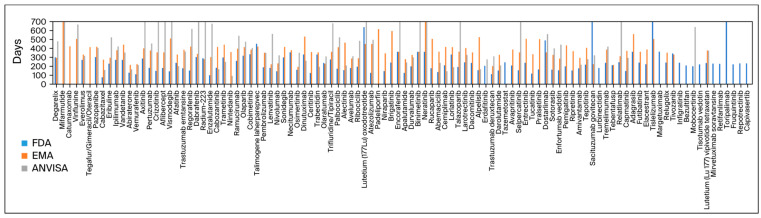
Approval time of every drug approved by the three agencies.

Figure 2 exhibits all drugs approved and their analysis times.

**Figure 3:**
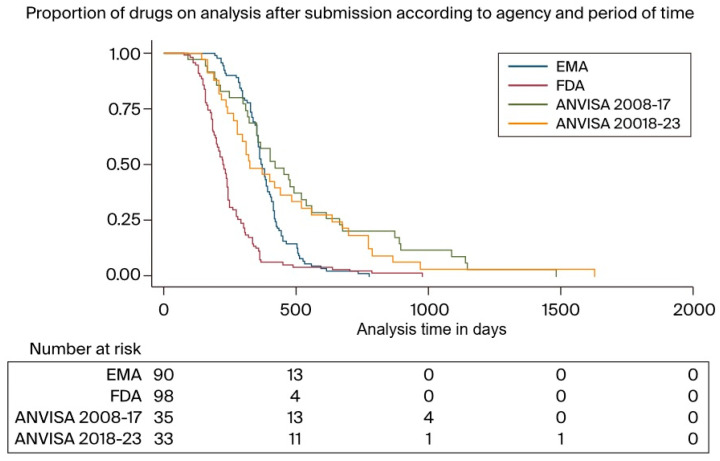
Proportion of drugs under analysis after submission according to agency and period.

The analysis period was also conducted across two distinct periods: from 2008 to 2017 and from 2018 to 2023. For both the FDA and the EMA, the analysis times during these periods were similar, irrespective of the timeframe. For the EMA, the median review time from 2008 to 2017 was 378 days (IQR: 303–420); in comparison, from 2018 to 2023, this figure stood at 364 days (329–416) (*p* = 0.8323). For the FDA, these figures stood at 215 days (154–300) and 223 days (171–245) across the two periods, respectively (*p* = 0.3320). Notably, the review times at ANVISA were 326 days (239–656) from 2008 to 2017 and 420 days (range: 309–664) from 2018 to 2023 (*p* = 0.3293). Figure 3 displays the proportion of drugs still under analysis after submission according to agency and when separating the ANVISA data by period as well.

### 3.4. Approval Dates

The FDA was found to be the agency that predominantly provided the first approval for the drugs in question. Among the drugs approved by at least two agencies, 11 were first approved by the EMA, whereas none were first approved by ANVISA. The remaining drugs were first approved by the FDA. When comparing EMA and ANVISA data, from a total of 67 drugs that were approved by both agencies, 10 were first approved by ANVISA.

When comparing data for the EMA to the FDA, the date of approval was 185 days (59–319) later, 558 days (278–957) later when comparing ANVISA to the FDA, and 435 days later (158–918) when comparing ANVISA to the EMA.

## 4. Discussion

The process of approving a new drug is multifaceted, involving stages dedicated to evaluating both the safety and efficacy of the medication, as well as the quality of evidence provided by the manufacturer. It is to be expected that the time for evaluation and issuance of a verdict vary among different regulatory agencies, depending on their internal processes and when the manufacturer submits their documents to the relevant committees.

The development of oncological therapies stands out due to the significant proportion of expedited approvals granted by both the FDA and the EMA [16,17]. These pathways, such as FDA accelerated approval and EMA conditional marketing authorization, enable earlier approval of medicines addressing unmet medical needs based on surrogate endpoints that are expected to predict clinical efficacy in later confirmatory trials.

In Europe, conditional marketing authorization is valid for one year and is subject to annual renewal to ensure that market authorization holders meet specific obligations within predefined timelines. In contrast, FDA accelerated approval does not undergo yearly review; however, confirmatory trials must be finalized within a specified timeframe.

A significant repercussion of early therapy approval is the uncertainty surrounding its appropriate use and overall benefit. For instance, when a drug is approved before the publication of the pivotal registration clinical trial, clinicians face a scarcity of comprehensive information regarding its safety and efficacy. This uncertainty has the potential to compromise patient safety in terms of therapy selection and toxicity management [2].

Moreover, drugs approved via expedited pathways may lack randomized data and/or rely on surrogate endpoints, leading to ambiguity regarding their overall clinical benefit [18]. Some drugs approved through accelerated pathways have been withdrawn from the market after confirmatory trials failed to demonstrate any benefit, highlighting the risk of relying on preliminary data. Additionally, confirmatory trials are often delayed and, in some cases, not completed at all [19,20].

The balance between shortening approval times, ensuring oncological patients receive therapies earlier, and ensuring treatments are safe and efficacious is delicate. Concerns about the efficacy of oncology therapies authorized by accelerated review and surrogate endpoints have been raised, as only 20% of drugs approved with a surrogate endpoint later show improvement in overall survival [21]. In addition, the results of previous studies have shown that drugs that receive accelerated approval by the FDA are twice as likely to receive a black box warning (defined as known serious risk) or be withdrawn from the market [22,23].

Although there are criticisms to be made regarding expedited programs, the results of our study demonstrate that the vast majority of drugs are approved by all three agencies, with the question being one of “when” rather than “if” they will be approved. Therefore, it cannot be concluded that agencies with longer review times and fewer approvals are being more careful; it may just be the case of not being fast enough.

Our data show that ANVISA takes a median of 73 days to initiate the analysis process compared to 22 days for the EMA, indicating a shortage of workforce capacity to begin the relevant processes. Another factor that may have influenced ANVISA’s analysis time is that it only became a member of the International Council for Harmonization of Technical Requirements for Pharmaceuticals for Human Use (ICH) in 2016, and it was accepted as a member of the Management Committee in 2019; therefore, pharmaceutical companies themselves face greater difficulty in submitting the necessary documents for ANVISA’s review due to the need for different formatting and taking longer to respond to ANVISA’s queries. Much of the delay in dossier analysis is due to the industry’s delayed responses to ANVISA’s inquiries [24,25].

At the end of 2017, ANVISA issued two board collegiate resolutions aimed at expediting drug approvals: Resolutions 204/2017 and 205/2017. Resolution 204/2017 establishes “Priority Review” standards for products that meet criteria such as rare, neglected, or emergent diseases without defined treatment alternatives. Resolution 205/2017 establishes new procedures for clinical trials, the certification of good manufacturing practices, and the registration of new drugs. For the priority category, the analysis must be completed in 120 days instead of 365 [25,26,27].

Another procedure established by ANVISA through Service Guideline #45 of 2018 is the creation of “detailed or optimized” registration analysis. This optimized approach may be adopted when the product registration request corresponds to a medication already approved by the FDA and EMA for the same indication, with the same dosage information, precautions, and adverse events listed on the label, and when all analysis documents from the FDA and EMA are available. This approach leverages the internal processes of other agencies to expedite its own, relying on the quality of analysis conducted by others [28].

The results of our analysis show that between 2008 and 2017, the time spent on reviews was similar for both the FDA and the EMA. However, the difference compared to ANVISA was significantly shorter by almost 100 days, although it did not reach statistical significance.

Project Orbis is a global collaborative review initiative that aims to accelerate patient access to innovative cancer therapies in multiple countries, led by the Oncology Center of Excellence at the FDA. Through Project Orbis, simultaneous regulatory submission, review, and action is facilitated between partner countries, including Australia, Brazil, Canada, Israel, Singapore, Switzerland, and the United Kingdom, and it has approved several new oncological therapies. The project’s goals are to increase efficiency and consistency in the regulatory process, avoid replication of the assessment of similar procedures, avoid wasting time and financial and human resources, and for countries to learn from each other’s experiences [8].

Earlier this year, ANVISA published a list of approvals from 2021 to 2023 through Project Orbis, and among the results are 6 new drugs for solid tumors, 2 for multiple myeloma, and 25 new indications for preapproved drugs for both solid and hematological tumors [29].

The differences in approval times between the FDA, ANVISA, and the EMA are influenced by a range of factors, including but not limited to the availability of resources, specific regulatory structures, and the socioeconomic context. Agencies continue to seek a balance between the need for quick access to innovative medicines and the importance of ensuring their safety and efficacy for the population. Therefore, understanding these differences is crucial for global stakeholders, including drug developers and public health policymakers.

It needs to be made clear that the fact that drugs have been approved by regulatory agencies does not mean that they will be readily available to the population. In the case of Brazil, there is an additional step for health insurers to approve oral medications, another for the public health system to propose incorporation, and yet another for the public health system to purchase such medications. As an example, the results of the CLEOPATRA trial were published on 12 January 2012 [25]. Pertuzumab was approved by the FDA on 8 June 2012, by the EMA on 13 December 2012, and by ANVISA on 27 May 2013. The drug was recommended for inclusion in the Brazilian public health system on 6 December 2017, and a purchasing contract between the Ministry of Health and Roche was signed on 3 July 2020 [30,31]. Thus, the above timeframe represents a seven-year gap in the availability of pertuzumab between the private and public sectors.

Our study has several limitations that must be addressed. First, we chose to exclude drugs used in the treatment of hematological neoplasms as they are outside our area of expertise; some drugs had to be excluded from the analysis due to different indications at the time of initial submission, such as gefitinib, whose first FDA approval was not contingent upon EGFR status, whereas approval from the EMA and ANVISA came years later, specifically for patients with EGFR mutations. We also sought the time from submission to analysis and approval solely for the first indication of each medication rather than for all indications listed in the label. This procedure was followed intentionally knowing that the initial approval is the most time-consuming and crucial step in making the medication available in different countries; however, we are aware that this study could be expanded to include other indications. Within the public databases, we were unable to find data on the waiting time between submission and the start of analysis for the FDA, and, in this study, we did not assess the quality of approvals or the number of approvals and indications that were subsequently withdrawn or modified. Subgroup analysis regarding drug classes and the primary site for which the drugs are intended were not performed because they would be underpowered for drawing any conclusion due to the great number of different classes and sites and, therefore, small numbers to analyze for any category. All of the data shown and all of the conclusions drawn are specific to the period in question (2008 to 2023); this timeframe, while extensive, may not fully capture long-term trends in regulatory policies and practices. Lastly, the factors influencing the speed of drug approvals, such as administrative processes, resource allocation, and differing regulatory requirements, were not extensively analyzed in the present study. These factors can significantly impact approval times and may vary greatly between regulatory bodies. The aim of future research should be to include the limitations listed above.

## 5. Conclusions

In summary, the impacts of delays in accessing oncological medications for cancer patients are a serious, urgent, and relevant issue in the field of oncology. These medications often represent the main line of defense against the progression of the disease and tumor growth, making them crucial for patient survival and quality of life. When delays occur in access, whether due to financial, bureaucratic, or logistical difficulties, patients face not only an obstacle to treatment but also a threat to their physical, emotional, and psychological well-being. The lack of promptness in administering these therapies can seriously compromise treatment response rates, leading to adverse consequences and negatively impacting patients’ prognosis [9,10].

The findings of our study indicate that the approval times for new drugs are considerably shorter for the FDA compared to those of ANVISA and the EMA. The FDA is typically the first agency to approve the drug in question and is often where the submissions are initiated. It is crucial to continue efforts to minimize and address these delays, helping to guarantee that patients have timely and sufficient access to the medications essential for their oncological treatment.

## Figures and Tables

**Table 1:** New drugs approved by one or more agencies from 2008 to 2023 for the treatment of solid tumors.

**FDA/EMA/ANVISA**	Abemaciclib	Dinutuximab	Pembrolizumab
	Abiraterone	Dostarlimab	Pertuzumab
	Afatinib	Durvalumab	Radium-223
	Aflibercept	Encorafenib	Ramucirumab
	Alectinib	Enfortumab vedotin	Regorafenib
	Alpelisib	Enzalutamide	Relatlimab
	Amivantamab	Eribuline	Ribociclib
	Apalutamide	Everolimus	Sacituzumab govitecan
	Atezolizumab	Ipilimumab	Sotorasib
	Avelumab	Larotrectinib	Talazoparib
	Axitinib	Lenvatinib	Tepotinib
	Binimetinib	Lorlatinib	Trabectidin
	Cabazitaxel	Lutetium (177Lu) oxodotreotide	Trametinib
	Cabozantinib	Lutetium (177Lu) vipivotide tetraxetan	Trastuzumab deruxtecan
	Capmatinib	Neratinib	Trastuzumab emtansine
	Cemiplimab	Nivolumab	Tremelimumab
	Cobimetinib	Olaparib	Trifluridine/tipiracil
	Crizotinib	Olaratumab	Vandetanib
	Dabrafenib	Osimertinib	Vemurafenib
	Darolutamide	Palbociclib	Vismodegib
	Degareliz	Pazopanibe	
**FDA/EMA**	Adagrasib	Necitumumab	Selpercatinib
	Avapritinib	Niraparib	Sonidegib
	Brigatinib	Pemigatinib	Talimogene laherparepvec
	Ceritinib	Pralsetinib	Tebentafusp
	Dacomitinib	Relugolix	Tislelizumab
	Elacestrant	Ripretinib	Tivozanib
	Entrectinib	Rucaparib	Tucatinib
	Futibatinib		
**EMA/ANVISA**	Mifarmutide	Nintedanib	Vinflunine
**FDA/ANVISA**	Erdafitinib	Mobocertinib	
**FDA**	Bezultifan	Lurbinectidin	Retifanlimab
	Capivasertib	Margetuximab	Tazemetostat
	Fruquintinib	Mirvetuximab soravtansine	Tisotumab vedotin
	Infigratinib	Repotrectinib	Toripalimab
**EMA**	Catumaxomab	Tegafur/gimeracil/oteracil	Padeliporfin

Abbreviations: FDA, Food and Drug Administration; EMA, European Medicines Agency; ANVISA, Brazilian Health Regulatory Agency.

**Table 2:** The primary site for which the new drugs were approved for their first indication. The total is 107 because 2 drugs were approved for more than one primary site.

Primary Site	n
Lung	24
Breast	15
Melanoma	12
Prostate	9
Kidney	7
Colorectal	4
Ovary, fallopian tube, and peritoneum	4
Thyroid	4
Agnostic	3
Bladder	3
Bile duct	3
Soft tissue sarcoma	3
Basal cell skin cancer	2
Gastric	2
GIST	2
Merkel cell carcinoma	2
Squamous cell skin cancer	1
Cervical	1
Uterine	1
Esophagus	1
Nasopharynx	1
Neuroblastoma	1
Osteosarcoma	1
Neuroendocrine	1

Abbreviation: GIST, gastrointestinal stromal tumor.

**Table 3:** Classes of new drugs.

Class	n
TKI	36
Monoclonal antibodies	21
Small molecule inhibitors	19
Cytotoxic	8
Hormonal agents	7
Antibody–drug conjugates	6
Immunomodulator	3
Radiopharmaceutical	3
Recombinant fusion protein	1
Viral therapy	1

Abbreviation: TKI, tyrosine–kinase inhibitor.

## Data Availability

The original data presented in the study are openly available from FigShare at https://doi.org/10.6084/m9.figshare.26300632.v1 (accessed on 23 July 2024).
